# Primary Anaplastic Lymphoma Kinase (ALK)-Negative Anaplastic Large Cell Lymphoma (ALCL) Presenting as Perforation Peritonitis: A Case Report and Management Principles

**DOI:** 10.7759/cureus.28738

**Published:** 2022-09-03

**Authors:** Vivek Sanker, Azeem Mohamed, Maanasi Pranala, Chaithra Jadhav, Varghese Tharakan

**Affiliations:** 1 General Surgery, NIMS Medicity (Noorul Islam Institute of Medical Science and Research Foundation), Trivandrum, IND; 2 General Surgery, Believers Church Medical College Hospital, Pathanamthitta, IND; 3 Pathology, Indira Gandhi Medical College & Research Institute (IGMCRI), Puducherry, IND

**Keywords:** resection, t-cell lymphoma, intestinal perforation, alk negative, alcl

## Abstract

Anaplastic large cell lymphoma (ALCL) has a characteristic feature that distinguishes it from the other types of non-Hodgkin lymphomas (NHLs) - the presence of a marker on its surface called CD30. It can be either cutaneous, systemic, or around breast implants. The systemic type of ALCL can be further classified based on the presence or absence of an abnormal protein, anaplastic lymphoma kinase (ALK), as ALK-positive or ALK-negative ALCL, respectively. We are presenting a case of a 35-year-old male who presented to the emergency department with an acute episode of abdominal pain. He underwent emergency laparotomy with ileal resection and anastomosis, as he was diagnosed with perforation peritonitis. Histological and immuno-histochemical reports of the specimen showed a lymphoproliferative lesion, and it helped reach the diagnosis of ALK-negative ALCL. ALK-negative ALCL can be diagnosed by using multidisciplinary investigation techniques, including radiological imaging, histopathological examination along with immunohistochemical staining. Prompt diagnosis helps in distinguishing ALK-negative ALCL from other lymphomas as well as solids tumors of the small bowel.

## Introduction

Anaplastic large cell lymphoma (ALCL) is a rare subtype of non-Hodgkin lymphoma with CD-30 positivity, which was described by Stein et al. in 1985 [1]. Anaplastic lymphoma kinase (ALK) is one of the members of the insulin receptor superfamily of receptor tyrosine kinases (RTKs). It was initially identified in constitutively activated oncogenic fusion forms, the most common being nucleophosmin (NPM) - ALK gene in anaplastic large-cell lymphomas. Based on the presence or absence of translocation involving the ALK gene to form a chimeric fusion protein NPM-ALK, ALCL is classified into ALK-positive and ALK-negative ALCL [2]. Most of the patients who are diagnosed with ALK-negative ALCL are seen to have involvement of the lymph nodes and extra-nodal sites such as skin, liver, or gastrointestinal tract and with a high tendency to invade the intestine. Primary intestinal involvement of CD-30-positive ALCLs is rare and needs a careful histochemical and pathological examination for a definitive diagnosis. The ALK-negative variant is found to have a greater prevalence among adults and with a slight male predominance. The median age at diagnosis is usually the fifth decade [3,4]. The ALK-negative subtype has an aggressive clinical presentation, shorter survival time, and thus an overall poor prognosis when compared with ALK-positive ALCL.

## Case presentation

We present a case of a 35-year-old male who was brought to the emergency department with complaints of diffuse abdominal pain of a duration of one week. On examination tenderness, rigidity and sluggish bowel sounds were present. The patient had no symptoms such as fever, night sweats, unexplained weight loss (B symptoms) and no peripheral lymph nodes were palpable. He had a past history of jaundice and pancreatitis. He was initially admitted to the medical gastro department for the evaluation of abdominal pain. Laboratory examination revealed a normal complete blood cell count with no leukocytosis (Table 1).

**Table 1:** Complete Blood Count (CBC)

Complete Blood Count	Patient Value	Reference Range
Hemoglobin (Hb)	10.5 gm/dL	11 – 17 gm/dL
Total Count (TC)	5980 cells/cumm	4000 – 11000 cells/cumm
Polymorphs	69.2%	50-80%
Lymphocytes	19.6%	25-50%
Monocytes	5.7%	2-10%
Eosinophils	4.2%	<5%
Basophils	1.3%	<2%
Platelet Count	2.68 lakhs/cumm	1.5 – 5 lakhs/cumm
Erythrocyte Sedimentation Rate (ESR)	60 mm/hr	0 – 20 mm/hr

**Figure 1:**
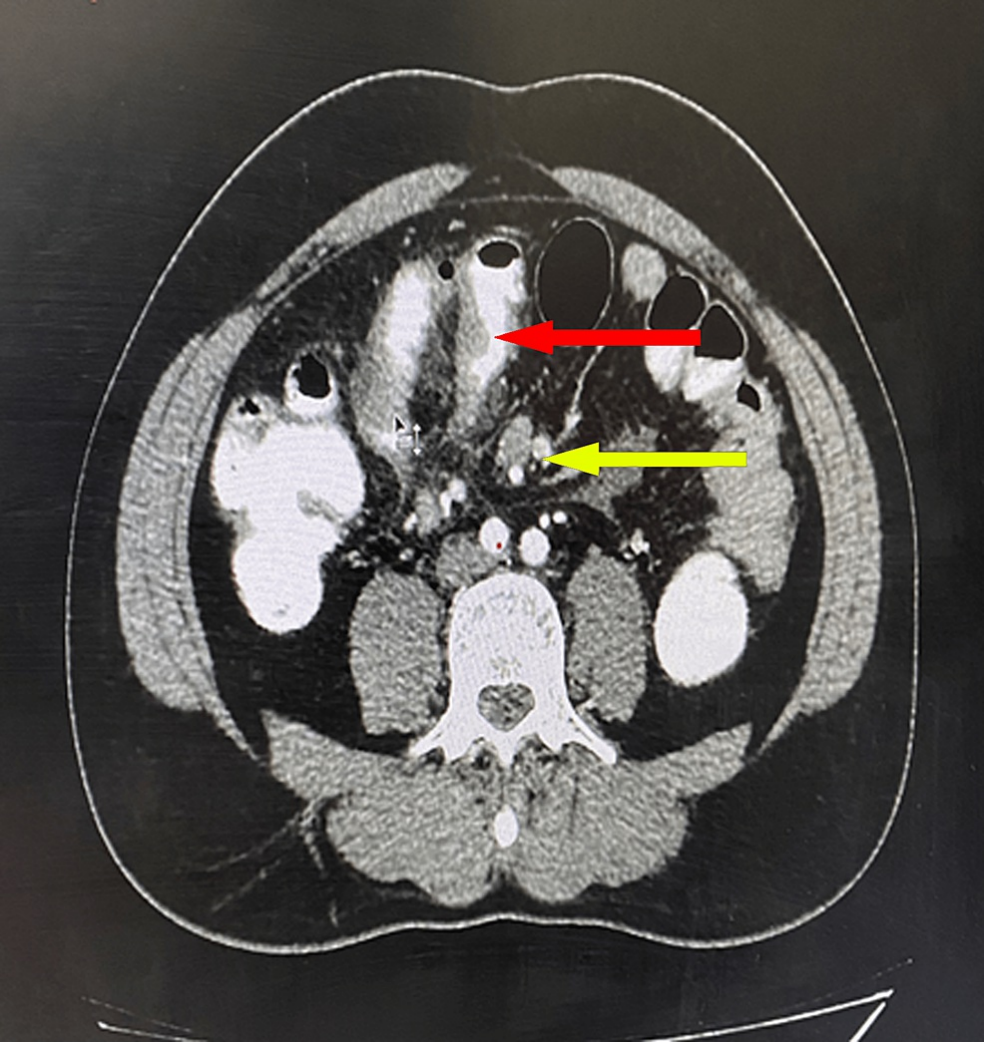
Post-contrast phase axial CT showing long segment edematous thickening of the mid ileal loop with surrounding fat stranding (shown in red arrow), multiple enlarged mesenteric lymph nodes (shown by the yellow arrow), and fluid and adjacent extra luminal air

Chest radiography was normal, and ultrasound of the abdomen and pelvis revealed hepatomegaly with grade I fatty changes along with multiple enlarged mesenteric lymph nodes in the umbilical region with mild adjacent fat stranding. Subsequently, contrast-enhanced computed tomography (CECT) scan of the abdomen showed pneumoperitoneum, ascites, long segment edematous thickening of the mid-ileal loop with surrounding fat stranding, fluid, and adjacent extra luminal air suggestive of infective/inflammatory etiology with contained perforation of the ileum (Figure 1).

**Figure 2:**
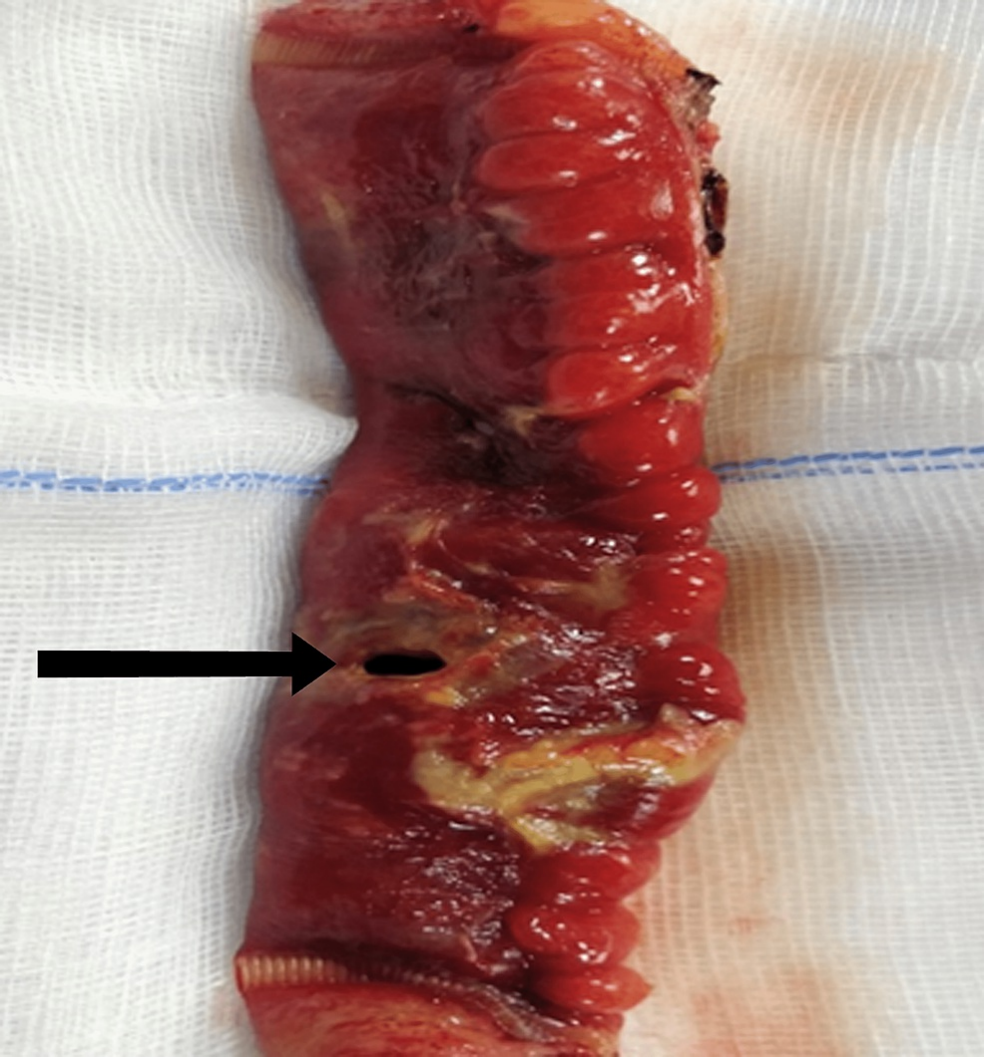
Gross photograph showing the resected specimen of ileum with the perforation site (shown in black arrow) and adjacent areas of necrosis

Surgical management was planned. After anesthesia clearance, he underwent an emergency exploratory laparotomy with ileal resection and anastomosis under general anesthesia. Intraoperative findings were consistent with an ileal perforation at 80 cm from the ileocecal junction with pelvic abscess, with adjacent bowel loops inflamed and multiple enlarged lymph nodes (Figure 2).

**Figure 3:**
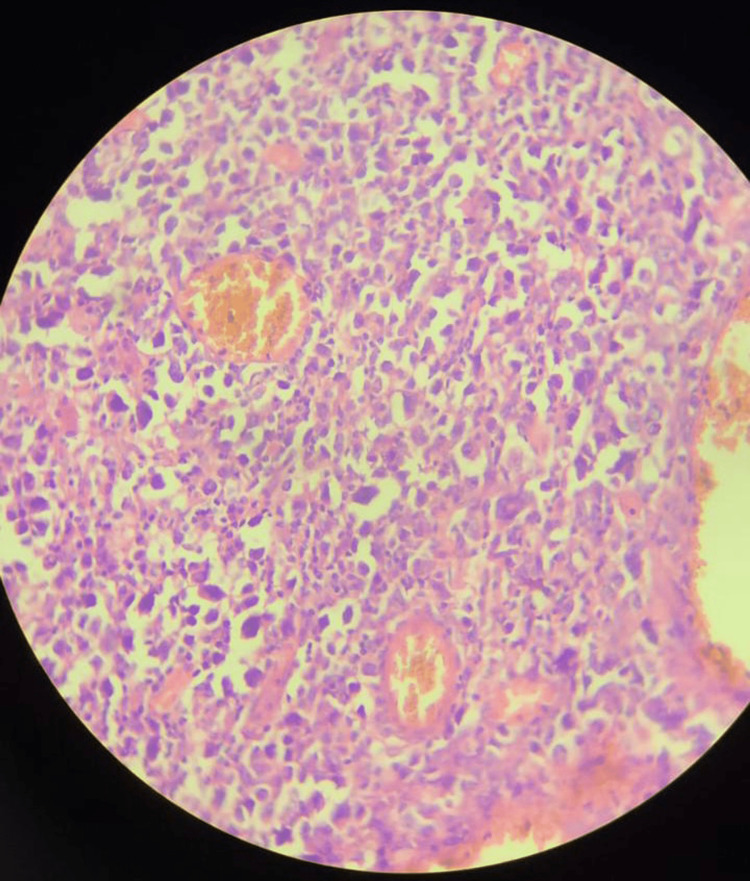
Biopsy of the ileum shows a submucosal lymphoid neoplasm composed of a polymorphous population of cells consisting of centrocytes, centroblasts, atypical lymphoid cells with the intended nucleus with basophilic cytoplasm and perinuclear clear Golgi zone seen. Hallmark cells with an eccentric horseshoe-shaped nucleus and occasional doughnut cells were noted. (H and E, 100x)

**Figure 4:**
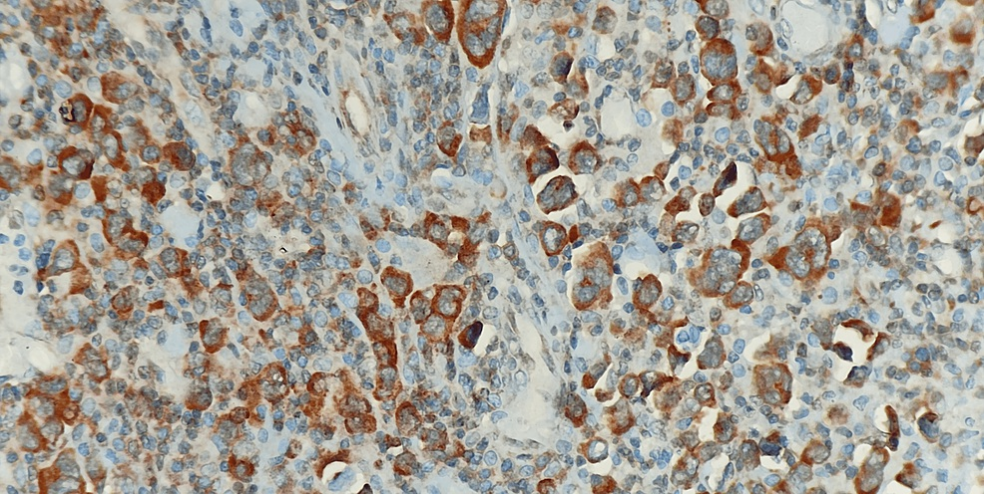
Immunostains showing the cells are strongly positive for CD 30 (400 x)

**Figure 5:**
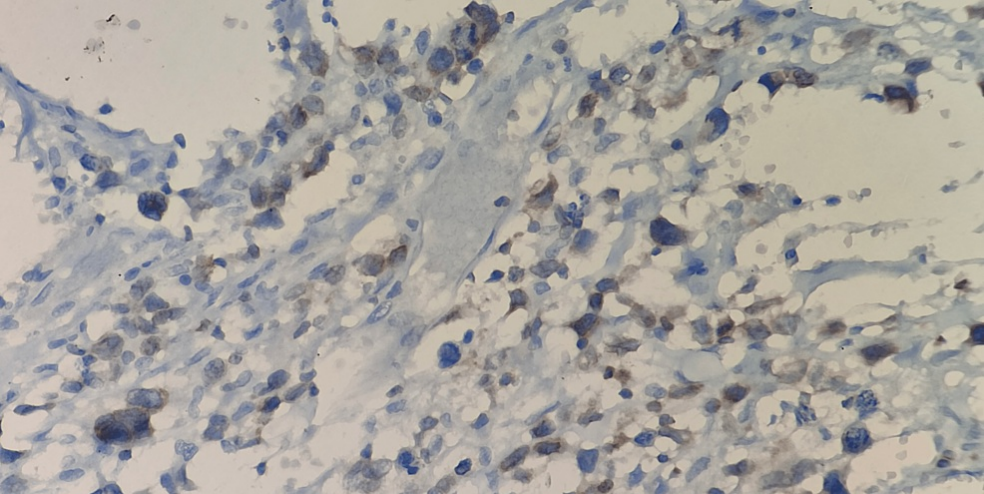
Immunostains showing the cells are positive for CD3 (400 x)

**Figure 6:**
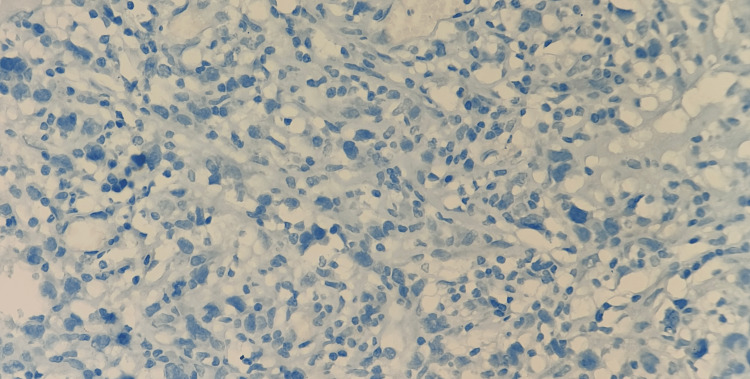
Immunostains showing the cells are negative for ALK (400 x)

The postoperative period was uneventful, and the patient got symptomatically better and hence was discharged. The histopathology report of the biopsy specimen revealed a lymphoproliferative lesion, possibly non-Hodgkin lymphoma. The section studied from the ileal mass shows a submucous lymphoid neoplasm with a polymorphous population of cells consisting of centrocytes, centroblasts, atypical lymphoid cells with the intended nucleus with basophilic cytoplasm, and perinuclear clear Golgi zone seen. Hallmark cells with an eccentric horseshoe-shaped nucleus and occasional 'doughnut cells' were noted. The neoplasm was seen extending transmurally and beyond the serosa (Figure 3). Immunohistochemical (IHC) tests on the formalin-fixed, paraffin-embedded surgical specimens were done by the Vantana platform method (Benchmark XT fully automated IHC stainer, Roche Diagnostics, Basel, Switzerland), prediluted, and the detection system horseradish peroxidase (HRP) polymer. The tumor cells were positive for CD-30 (Figure 4), CD-45, CD-3 (Figure 5), CD-8, and CD-56 and were negative for cytokeratin, epithelial membrane antigen (EMA), ALK (Figure 6), CD-15, CD-20, CD-5, and S-100.

Bone marrow aspirate showed mild lymphocytosis with no infiltration. Flow cytometry was not performed. He was diagnosed with a case of CD-30 positive primary intestinal ALK-negative ALCL and was advised to undergo chemotherapy after an oncology consultation. The patient is currently being treated with CHOEP regimen chemotherapy (Cyclophosphamide, Doxorubicin, Vincristine, Etoposide, and Prednisone), which is the standard chemotherapy regimen, and has successfully completed five cycles. Each cycle is given to the patient once every three weeks.

## Discussion

ALCL is an uncommon peripheral T-cell lymphoma and is a subtype of NHL with classic findings of large lymphocytes with nuclei that are horse-shoe shaped and a uniform expression of CD 30+ on the membrane [5]. Based on the translocation involving the ALK gene, there are two distinct subtypes. This differentiation is important because of clinical and prognostic differences. ALK-negative ALCL typically has a clinically aggressive presentation and an unfavorable prognosis compared to ALK-positive ALCL. ALK-positive systemic ALCL, ALK-negative systemic ALCL, and primary cutaneous ALCL (ALK-negative) are the three subtypes of ALCL based on ALK expression [6,7]. ALK-negative ALCL patients of systemic type have a worse prognosis than ALK-positive ALCL patients, with five-year survival rates of 49% and 70%, respectively [8]. The peak incidence of ALCL, ALK-negative is in adults (above 40 years) in contrast to ALCL, ALK+, which occurs more frequently in children and younger adults [9]. ALCL, ALK-negative cases mostly tend to involve the lymph nodes, and extranodal involvement is rare, usually limited to skin, bone, and soft tissues. Gastrointestinal involvement with ALK- ALCL is extremely rare [10,11].

CD-30 positivity is seen in ALCL, classic Hodgkin lymphoma, peripheral T-cell lymphoma, primary effusion lymphoma, diffuse large cell lymphoma, and plasmablastic lymphoma. The ALCL tumor cells also express EMA and other T-cell markers such as CD-3, CD-7, and CD-8 [12]. Cells are negative for CD-2, CD-20, CD-79a, CD-43, PAX5, Tdt, CD-138, CD-10, BCL-2, ALK, and MPO. In our case, tumor cells were positive for CD-30 (diffuse), CD-45 (LCA), CD-3, CD-7, CD-8, CD-56, and granzyme and negative for ALK-1, Cytokeratin (CK), EMA, CD-15, CD-20, CD-5, and S-100. CD-30 is an antigen that has a role in lymphocyte activation and is crucial for the diagnosis of classic Hodgkin lymphoma, ALCL, and embryonal carcinoma. The absence of Reed Sternberg (RS) cells and other B-cell markers such as CD-19, CD-20, CD-79a, CD-138, and PAX-5 helped rule out Hodgkin lymphoma. CD-30 is a cell surface receptor tumor necrosis factor and a lymphocyte activation antigen. The molecular basis for aberrant growth and cytokine expression is due to the over-expression of CD-30, which leads to the constitutive expression of nuclear factor Kappa B. The strong, diffuse, membranous, and Golgi-associated staining pattern of CD-30, CD-3 (pan T-cell marker) positive, granzyme positive along with the cell morphology helped make the diagnosis of ALCL [6]. CD-56 is a homophilic binding glycoprotein with a role in cell-cell adhesion. A study by Suzuki et al. showed that CD56 positivity in ALCL heralds poor prognosis irrespective of the ALK status [13].

In our patient, the tumor cells showed an absence of ALK expression on IHC, and the clinical presentation was similar to the classical clinical findings for ALK-negative systemic ALCL. The most important factors that determine the prognosis of patients include age, The Prognostic Index for T-cell lymphoma (PIT) scoring system, β2-microglobulin, and bone marrow infiltration [14]. The prognosis also depends on the expression of proteins involved in the regulation of apoptosis (caspase 3, Bcl-2, PI9) and that of CD-56 [13,15]. The first line of treatment for ALCL is chemotherapy, irrespective of the ALK status. CHOP (cyclophosphamide, doxorubicin, vincristine, prednisone) is the most commonly used regimen to treat systemic ALCL patients above 60 years of age and should abide by the CHOEP regime if less than 60 years of age [9]. The index patient successfully received five cycles of chemotherapy (CHOEP regimen) and is compliant with the current treatment regimen with no complications. Although it is difficult to predict which patients will have a positive response to the treatment regimen, induction chemotherapy is initially given, and some patients also receive additional consolidative autologous transplantation during the first complete remission. The genetic heterogeneity of the disease may be the reason for the variability in overall survival rates and treatment responsiveness and therefore, further studies are warranted to ascertain which patients might benefit from more aggressive treatment modalities.

## Conclusions

Anaplastic large cell lymphoma (ALCL) is a very rare tumor, which is classified under non-Hodgkin lymphoma. The ALK-negative variant of ALCL, which our patient was diagnosed with, is still more uncommon to come across in clinical practice. From a clinician’s point of view, since most clinicians do not consider ALCL as a differential diagnosis for mass abdomen cases, our case indicates that it should be suspected in a patient of mass abdomen with positive histopathological findings, which helps in the confirmation of the diagnosis as lymphoma. Thus, early detection leads to appropriate treatment and, finally, a better quality of life.
